# Functional MRI for the prediction of treatment response in head and neck squamous cell carcinoma: potential and limitations

**DOI:** 10.1186/s40644-016-0080-6

**Published:** 2016-08-19

**Authors:** Ann D. King, Harriet C. Thoeny

**Affiliations:** 1 Department of Imaging and Interventional Radiology, Faculty of Medicine, The Chinese University of Hong Kong, Prince of Wales Hospital, 30-32 Ngan Shing Street, Shatin, New Territories, Hong Kong S.A.R. China; 2 Department of Radiology, Neuroradiology and Nuclear Medicine, Inselspital, University of Bern, Freiburgstrasse 10, 3010 Bern, Switzerland

**Keywords:** MRI, DWI, DCE, MRS, Head and neck, Cancer, Squamous cell carcinoma, Radiotherapy, Chemotherapy, Response

## Abstract

Pre-treatment or early intra-treatment prediction of patients with head and neck squamous cell carcinomas (HNSCC) who are likely to have tumours that are resistant to chemoradiotherapy (CRT) would enable treatment regimens to be changed at an early time point, or allow patients at risk of residual disease to be targeted for more intensive post-treatment investigation. Research into the potential advantages of using functional-based magnetic resonance imaging (MRI) sequences before or during cancer treatments to predict treatment response has been ongoing for several years. In regard to HNSCC, the reported results from functional MRI research are promising but they have yet to be transferred to the clinical domain. This article will review the functional MRI literature in HNSCC to determine the current status of the research and try to identify areas that are close to application in clinical practice. This review will focus on diffusion-weighted imaging (DWI) and dynamic contrast-enhanced MRI (DCE–MRI) and briefly include proton magnetic resonance spectroscopy (^1^H-MRS)and blood oxygen level dependent (BOLD) MRI.

## Background

Head and neck squamous cell carcinoma (HNSCC) is the most common cancer in the head and neck. The two main modalities of treatment with curative intent for patients with advanced stage HNSCC are surgery or chemoradiotherapy (CRT). Over the past decade the non-surgical approach has become more popular because of the better organ preservation, but not all HNSCCs respond to CRT and approximately 25–30 % of patients will fail treatment at local or nodal sites in the head and neck. The successful pre-treatment identification of patients with resistant tumours in the head and neck would allow the CRT regimes to be modified or changed to a surgical approach. In addition intra-treatment scanning is already under evaluation to adapt radiotherapy (RT) fields to the changing size of the tumour [1] providing an opportunity to monitor early treatment response and adjust CRT regimes accordingly. Even if treatment is unaltered the identification of primary or nodal sites at risk of treatment failure would allow those sites to be targeted for more aggressive post-treatment investigation, so that residual cancers can be identified while they are still amenable to salvage surgery.

Computed tomography (CT), fluorine-18-fluorodeoxyglucose-positron emission tomography (^18^FDG-PET)/CT and magnetic resonance imaging (MRI) are used to identify resistant HNSCCs. All three imaging modalities are well established in routine clinical practice for staging head and neck cancer, planning treatment and assessing post treatment response. Each imaging modality has its own merits the discussion of which is beyond the scope of this review. This review will focus instead on MRI which produces both anatomical and functional information, and is ideally suited to serial scanning, including repeat scanning early in the course of treatment. In common with all anatomical-based imaging, the anatomical MRI sequences have limitations in predicting treatment response and in monitoring response, hence the current interest in adding functional-based MRI sequences to the standard MRI examination protocol.

Functional MRI head and neck cancer research has been ongoing for more than 10 years, but there are many challenges still to be faced before functional MRI can be transferred from the research to the clinical domain. This article will review the current status of functional MRI techniques in HNSCC for pre-treatment and early intra-treatment prediction of response, with the emphasis on diffusion-weighted imaging (DWI) and dynamic contrast-enhanced MRI (DCE–MRI) and a brief inclusion of proton magnetic resonance spectroscopy (^1^H-MRS) and blood oxygen level dependent (BOLD) MRI.

## Main text

### Functional MRI in HNSCC

Functional MRI is a technically challenging examination to perform on patients with head and neck cancer, because acquisition is hampered by artefacts, including susceptibility artefacts and those related to movement from coughing, swallowing and breathing. Moreover, the acquisition parameters have yet to be standardized across centres, and even when similar protocols are used reproducibility can be problematic. Once the data has been acquired there is variability in the methods of analysis. This includes issues such as whether to analyse the primary tumour or nodal metastases, where to place the regions of interest (ROI), and whether to use programs that are developed “in-house” or by produced by the different vendors data. Data obtained from the entire tumour volume is more representative of the tumour, compared with data obtained from small ROIs within the tumour or from a ROI encompassing the largest cross-sectional tumour area, but even so most studies have reported only the mean value of the functional parameter, which does not fully reflect tumour heterogeneity. To analyse tumour heterogeneity pixel by pixel analysis is being used because it enables functional data to be displayed as a histogram and provides additional parameter measures such as percentiles, volumes of parameters in a defined range, and those measures that reflect the shape of the distribution curve. The drawbacks of these sophisticated methods of analysis are that they are more technically challenging and it is difficult to isolate a single functional parameter measure that consistently predicts outcome.

Furthermore, an array of treatment endpoints has been used to analyse the predictive value of each functional MRI parameter. These endpoints include a change in tumour size post-treatment which may not necessarily predict clinical outcome, and tumour relapse in the head and neck which preferably is assessed over a follow-up period of at least 2–3 years when the vast majority of resistant HNSCCs are known to recur. Relapse at specific primary or nodal sites in the head and neck are valuable endpoints for studies that are designed to influence intra-treatment adaptive therapies or identify sites at risk of post-treatment residual disease. These are the endpoints that are of greater interest to the radiologist and are especially valid for head and neck MRI. However, many studies use more general endpoints such as relapse at any site including distant sites, or survival including overall, disease-specific, disease-free, progression-free and local relapse-free survival.

In summary, for any given functional MRI technique variable methods are used for image acquisition, image analysis and data correlation. Literature comparison is therefore not straightforward because a range of different functional parameter measures, and their respective thresholds, have been reported as predictors of outcome based on a range of different treatment outcomes. It is also worth noting that many functional MRI studies do not incorporate non-radiological confounding factors into the analysis, such as the human papilloma virus (HPV) status, which is an important independent predictor of the HNSCC outcome. These issues will be further explored below in relation to each functional modality.

### Diffusion-weighted imaging (DWI)

#### Pre-treatment DWI for prediction of treatment response

**Fig. 1:**
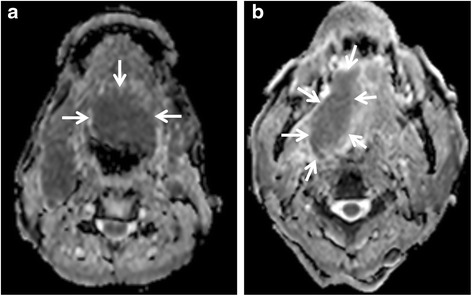
**a & b**. Pre-treatment DWI for prediction of CRT response. **a** Pre-treatment ADC map of a squamous cell carcinoma of the tongue (*arrows*) with a low ADC (0.73 × 10^−3^ mm^2^/s) in a tumour with local control; **b** Pre-treatment ADC map of a squamous cell carcinoma of the tongue (arrows) with a high ADC (1.10 × 10^−3^ mm^2^/s) in a tumour with local failure

HNSCCs with high stromal content, low cellularity (lower proliferation), micronecrosis and negative HPV status are associated with resistance to treatment and poor outcome. These tumour characteristics decrease diffusion restriction of water molecules in HNSCC, as reflected by higher apparent diffusion coefficients (ADC) [2–4]. Therefore, it is hypothesised that high tumour ADC is a predictor of poor outcome. However, an argument could also be made for a low ADC being a predictor of poor treatment response, based on the observation that poorly differentiated HNSCCs have lower ADCs than well differentiated HNSCCs [5–8] and poorly differentiated HNSCCs are more likely to spread to regional and distant sites. Currently the reported DWI results seem to favour the high mean ADC as the predictor of poor treatment response [9–14] (Fig. 1a, b). Three studies have evaluated tumour sites in the head and neck and found that primary tumours with primary failure [10, 11] and nodal metastases with nodal failure [9] showed significantly higher mean pre-treatment ADCs than tumours with control at those sites. These studies were able to identify cut-off thresholds but the reported thresholds have a broad range of > 0.79 to 1.1 × 10^−3^ mm^2^/s, two obtained from b values ranging from 0–1000 s/mm^2^ [9, 11] and the other from 300–1000 s/mm^2^ [10]. Another three studies included data from distant site relapse or death in the calculation of clinical outcome, and these studies found high ADC values were predictors of disease relapse at any site [12], rate of 3 year regional control (ADC threshold of 1.14 × 10^−3^ mm^2^/s) [13], and disease free survival (ADC threshold of 1.51 × 10^−3^ mm^2^/s) [14]. Four further studies also found higher ADC values [15–17] or higher D values (pure diffusion values from intravoxel incoherent motion, (IVIM)) [18] in unfavourable compared to favourable outcome groups, but the results of these four studies did not reach significance for predicting failure at head and neck sites [15, 16, 18] or failure at any site in the body [17]. Moreover, a study by Nakajo et al. [19] found the opposite finding to the studies above, a high ADC being a predictor of better outcome based on two-year disease free survival.

Most DWI studies exclude the macroscopic necrotic regions of a tumour from the analysis, but HNSCCs are still heterogeneous tumours and the use of the mean ADC value has limitations. To overcome these limitations further research is required to find ways to analyse different populations of tumour cells within HNSCC. This includes using parameters such as ADC min [20] with one recent study by Preda et al. [21] finding a high ADC min, obtained from one standard deviation below the mean, was a significant predictor of poor disease-free-survival.

There are three further variables that need to be considered when comparing the DWI results in the literature. The first variable relates to the head and neck tumour site chosen for data acquisition. A study by Noij et al. [14] in the same patient population found that data acquired from nodal sites were predictive of outcome, whereas data acquired from primary sites were not. The second variable relates to the choice of b values for DWI acquisition and analysis. Recent studies have shown that mean ADCs obtained from high b value ranges of 300/500–1000 s/mm^2^ (“pure” diffusion) are more predictive of treatment response than mean ADCs obtained from low b value ranges of 0–100/300 s/mm^2^ (“perfusion-related” diffusion) [10, 12]; but even within the higher range one study has shown mean ADCs are predictive at 0–750 s/mm^2^ but are not predictive at b 0–1000 s/mm^2^ [14]; and another study has shown mean ADCs are predictive at b 0–2000 s/mm^2^ but are not predictive at b 0–1000 s/mm^2^ [22]. The third variable relates to the variability of ADC values with different MRI systems and sequences that has been reported in some studies [23]. Therefore, further research includes identifying the best tumour sites in the head and neck to select for functional imaging, the optimum range of b values for DWI acquisition and analysis, and the optimum ADC parameters and thresholds.

#### Intra-treatment DWI for prediction of treatment response

**Fig. 2:**
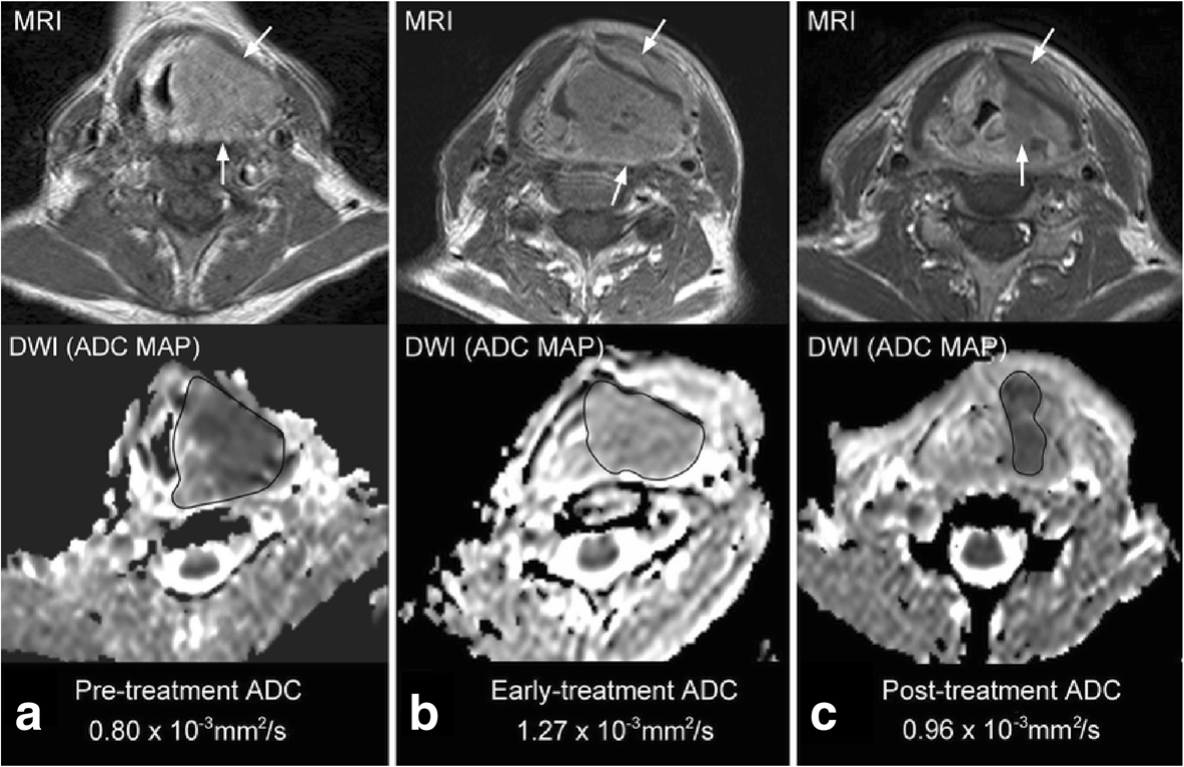
**a**, **b & c**. Change in DWI over treatment for prediction of CRT response. ADC maps of a hypopharyngeal SCC (*arrows*) with local failure; **a** Pre-treatment, **b** two weeks intra-treatment, **c** 6 weeks post-treatment. The ADC shows the expected initial early rise in ADC but there is a later fall in ADC (believed to be due to repopulation of tumour cells). {Image reproduced from: King AD, Mo FK, Yu KH, et al. (2010) Squamous cell carcinoma of the head and neck: diffusion-weighted MR imaging for prediction and monitoring of treatment response. Eur Radiol 20:2213-20.}

As response adapted therapy becomes more widespread in cancer management there will be greater interest in performing intra-treatment scanning. A rise in ADC occurs in the first few weeks after the start of HNSCC treatment and there is now evidence to show the percentage (%) rise in ADC is a predictor of treatment response. Four studies have shown a smaller % rise in the mean ADC 1–3 weeks after the start of treatment in patients with disease failure compared to those with disease control [9, 15, 16, 24]. Three of these studies found thresholds of < 14–24 %, were predictors of treatment failure in head and neck sites using clinical outcome data with follow-up for at least 2 years [15, 16, 24]. This approach is promising because the % ADC change could be more reproducible across centres than absolute ADC values, but further research is required to confirm the optimum % change threshold for clinical use. In the future serial DWI of HNSCC will provide an opportunity to closely monitor response not only in the early treatment phase but throughout treatment. In this regard it has been observed that after the initial early ADC rise a subsequent ADC fall predicts locoregional failure, it is postulated that this ADC fall is caused by the repopulation of cancer cells [25] (Fig. 2a-c). However, it is very important to exclude frankly necrotic areas from ADC analysis because sterile areas of necrosis may take longer to resolve than solid areas, and in the interim the necrosis may become organised and show a fall in the ADC value. Therefore, it is critical to assess the ADC map in conjunction with the anatomical-based MRI sequences to identify sites of necrosis that need to be excluded from ADC analysis Furthermore with time the development of mature scar tissue may also decrease the ADC value [26].

**Fig. 3:**
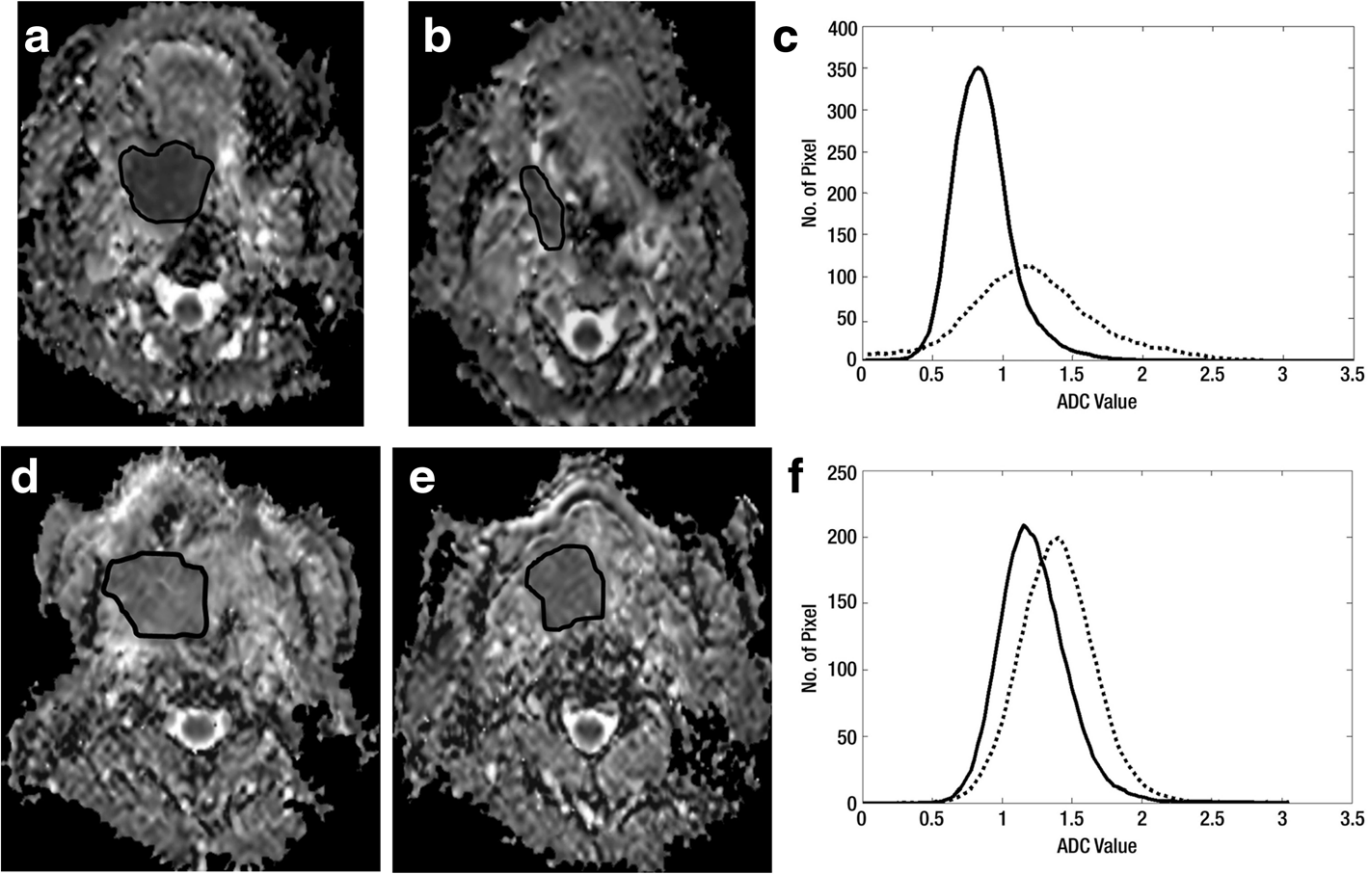
**a-f**. Analysis of the early intra-treatment ADC distribution curve for prediction of CRT response. ADC maps of a tonsil SCC (ROI) with local control; **a** pre-treatment, **b** two weeks intra-treatment, **c** ADC distribution curve pre-treatment (*solid line*) and two weeks intra-treatment (*dashed line*). ADC maps of a tongue SCC (ROI) with local failure; **d** pre-treatment, **e** two weeks intra-treatment, **f** ADC distribution curve pre-treatment (*solid line*) and two weeks intra-treatment (*dashed line*). The ADC distribution curve of the tumour with local failure shows less movement to the right side, (smaller increase in ADC mean) and a high skewness (skewness has fallen, but the short tail still remains on the positive left side) and the curve still has an acute peak with a high ADC kurtosis (kurtosis has fallen only slightly and remains high). {reproduced with permission of Radiology and [King AD, Chow KK, Yu KH, et al. Head and neck squamous cell carcinoma: diagnostic performance of diffusion-weighted MR imaging for the prediction of treatment response. Radiology. 2013 Feb;266:531–538]}

More sophisticated methods of intra-treatment analysis which take into account the heterogeneity of treatment induced-changes within a tumour have shown promising results. One such method used parametric response maps to monitor the change in the volume of ADC subsets [27] while another analysed the shape of the ADC distribution curve two weeks after the start of treatment and found tumour sites with local control had curves skewed towards the high ADC values and were more heterogeneous with a broader peak, compared to tumour sites with local failure (Fig. 3a-f) [15]. Furthermore, DWI may have an important role in the evaluation of treatment response of the new molecular targeted agents such as the antiangiogenic agent Bevacizumab which is a monocolonal antibody that inhibits vascular endothelial growth factor (VEGF). This factor is present in HNSCCs and its inhibition could be valuable in the deintensification of CRT regimes in HPV related tumours. Drug trials of these agents in combination with CRT regimes are underway in HNSCC, but is known already from research into gliomas that Bevacizumab may produce a pseudo-response on imaging, whereby a reduction in the enhancing component of the tumour and surrounding oedema may mask progression of the underlying tumour. In this scenario the presence of persistent or increasing restriction of diffusion may be a better marker of disease progression.

#### Summary of the role of DWI

DWI research points to high pre-treatment mean ADC and a low % rise in ADC early intra-treatment being indicators of poor outcome. Intra-treatment scanning is likely to become more important in clinical management and one of the main advantages of DWI over DCE or PET/CT is that it does not require an injection of gadolinium or FDG. In this regard the early % change in mean ADC for predicting response at tumour sites in the head and neck is one of the most promising clinical applications for DWI, once a cut-off threshold has been confirmed. However, further work is needed to improve quality assurance across different MRI scanners, standardise the DWI protocol, especially in relationship to the choice of b values, and develop more sophisticated methods for analysing heterogeneous treatment induced changes. In addition, ongoing technological advances continue to reduce susceptibility and motion artefacts associated with DWI in the head and neck.

### Dynamic Contrast-Enhanced MRI (DCE-MRI)

#### Pre-treatment prediction

**Fig. 4:**
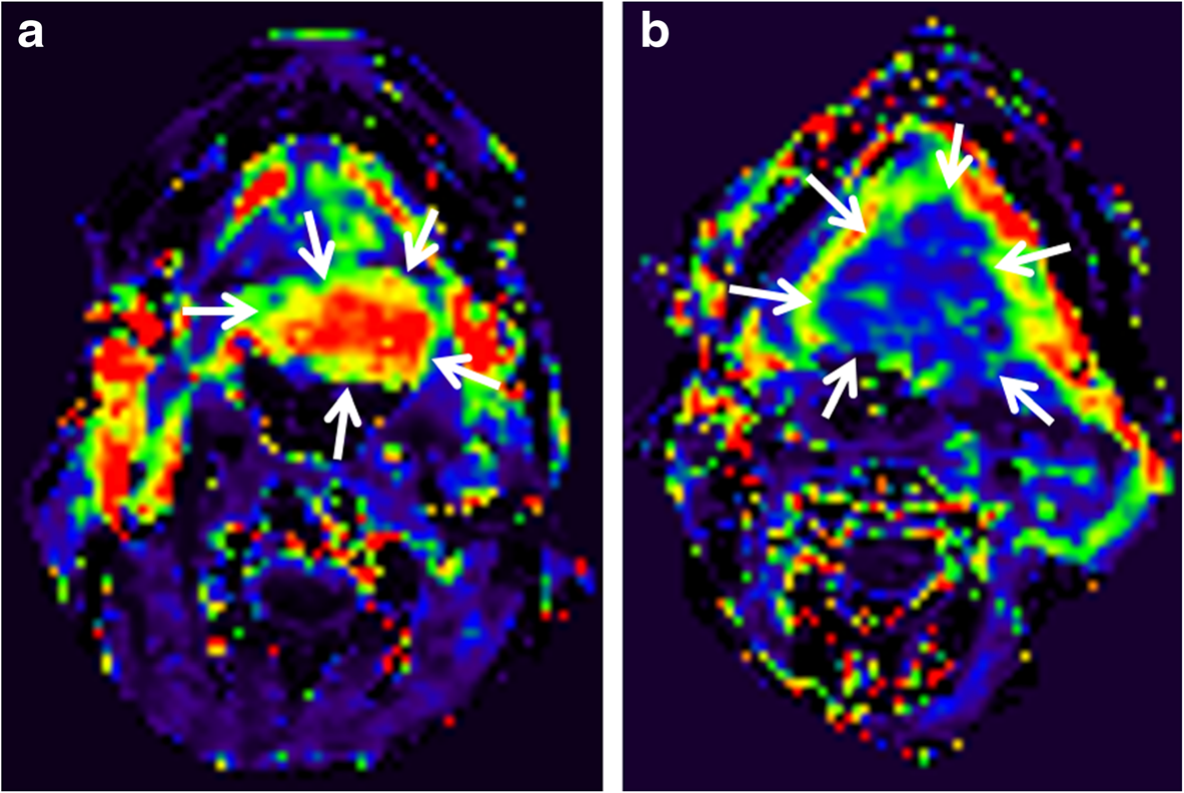
**a & b**. Pre-treatment DCE-MRI for prediction of CRT response. **a** Pre-treatment K^trans^ map of a squamous cell carcinoma of the tongue (*arrows*) with a high K^trans^ (0.87 min^−1^) in a tumour with local control; **b** Pre-treatment K^trans^ map of a squamous cell carcinoma of the tongue (*arrows*) with a low K^trans^ (0.29 min^−1^) in a tumour with local failure

Vascular HNSCCs are thought to have a better treatment response compared to less vascular HNSCCs (Fig. 4a, b), because of better delivery of the chemotherapeutic agents and greater radiosensitivity. On the other hand vascular tumours may have a poorer outcome because it is thought they have greater metastatic potential. Vascular-related parameters of HNSCC can be studied using DCE-MRI which evaluates the passage of a standard gadolinium-based MRI intravenous contrast agent in the intravascular and extravascular extracellular space of the tumour. These can be measured using semiquantitative techniques which analyse the time-signal intensity curve, or quantitative techniques such as the pharmacokinetic models (PKM) described by Tofts [28]. Compared with DWI, DCE provides more potential functional parameters for analysis but the data acquisition is more challenging, especially when using PKMs, which have many technical problems that have yet to be ironed out, including the accurate measurement of the arterial input function. Currently the K^trans^ min^−1^ (the volume transfer constant between the blood plasma and extracellular extravascular space) obtained from PKM appears to be one of the most consistent DCE parameters to show a correlation with outcome. Most studies suggest high K^trans^ values in tumours with a good response [13, 17, 29–32], one of which acquired functional data from the primary tumour sites [29], while the other five evaluated metastatic nodes. However, various outcome measures were used and clinical thresholds were reported in only three of these studies ranging from > 0.41 to 0.84 min^−1^[13, 29, 31]. Moreover, two pre-treatment DCE-MRI studies were unable to show any correlation between K^trans^ [33] or blood volume and blood flow [34] and outcome. Therefore, at present the data for pre-treatment DCE-MRI seems to be insufficient to allow translation to clinical practice.

#### Intra-treatment prediction

There is less reported research related to intra-treatment monitoring using DCE-MRI compared to DWI possibly because DCE-MRI requires an intravenous injection of a contrast agent. There is some early work in tumour xenografts to suggest a rise in tumour K^trans^ may occur over the first few days of treatment [35], and in human subjects to suggest a rise in plasma flow at two weeks [36]. It is postulated that an early rise in K^trans^ is due to damage to the blood vessels causing them to temporarily become leakier, which potentially could increase the delivery of chemotherapeutic agents into the tumour. Indeed a few reports suggest a fall in K^trans^ & area under the gadolinium concentration-time curve (AUGC) is associated with poor overall survival [37], while an early rise in blood volume is associated with local control [34]. However, further research is required, especially as results may be influenced by different therapeutic modalities and regimes including the use of anti-angiogenic agents.

#### Summary of the role of DCE-MRI

DCE-MRI is an even more challenging functional technique to perform in patients with HNSCC than DWI, and it has a greater range of methods and functional parameters for analysis. Research points to better assess treatment responses in tumours with higher vascular-related parameters HNSCCs, a high pre-treatment K^trans^ being predictive of a good response, although clinically useful thresholds have yet to be established. Intra-treatment monitoring is promising but still at a relatively early stage of research.

### Proton Magnetic Resonance Spectroscopy (^1^H-MRS) and Blood Oxygen Level Dependent (BOLD) MRI

^1^H-MRS was one of the first functional techniques to be assessed in the characterisation of tumours in the head and neck, but it remains a challenging technique in this region of the body, and in regard to pre-treatment prediction and intra-treatment monitoring of response there is a paucity of a research in this area when compared to DWI and DCE-MRI. One in vitro study of tumour specimens by Bezabeh et al. [38] has shown significantly elevated pre-treatment choline-to-creatine ratios in a poor response group, but these findings could not be corroborated in an in vivo human study using choline-to-creatine ratios as well choline-to-water ratios [39]. This latter study was also unable to show any predictive value for the 2 week intra-treatment scan [39].

Tumour hypoxia reduces the effectiveness of CRT and is associated with an unfavourable outcome in HNSCC [40]. Tumour hypoxia may be evaluated by different techniques which include non-invasive in vivo MRI using BOLD which relies on the paramagnetic effect of blood deoxyhaemoglobin to decrease the signal intensity on T2* images. BOLD identifies changes in tumour oxygenation while breathing oxygen or carbogen, which increases diamagnetic oxyhaemoglobin leading to an increase in the signal intensity within the tumour on T2* images. Although BOLD imaging has been shown to be feasible [41, 42] and T2* quantitative imaging may be sensitive and reproducible [43] in HNSCCs the predictive value of BOLD MRI currently is unknown.

## Conclusions

Functional MRI research has identified parameters that have the potential to predict CRT response in patients with HNSCC either before the start of treatment or during a course of treatment. High ADCs from DWI and low K^trans^ from DCE-MRI in pre-treatment head and neck sites are associated with unfavourable treatment outcomes, but clinical thresholds have yet to be established and research into the optimum methods of data acquisition and analysis are still ongoing. In the future it is likely that intra-treatment scanning will become more important in clinical management allowing modification of CRT regimes or the cessation of ineffective treatments. In this regard DWI, which does not require an intravenous injection of an exogenous agent, is the most promising technique and current results suggest that those tumours with a low % rise in ADC early in the course of treatment are more likely to fail treatment than those tumours with a high % rise in ADC.

Future advances are likely to come from optimisation of acquisition protocols, the development of more sophisticated methods of analysis which take into account tumour heterogeneity and allow serial intra-treatment changes to be monitored, and multiparametric imaging combining not only the different functional MRI parameters but also PET parameters on the new PET/MRI systems. However, in order for functional MRI to make it into mainstream of cancer management it must to be reproducible across multiple centres. Working groups are looking into the quality assurance and standardization of methods, but more work is needed to set up acceptable guidelines as functional MRI evolves [44–46].

## Abbreviations

ADC, apparent diffusion coefficients; AUGC, area under the gadolinium concentration-time curve; CRT, chemoradiotherapy; DWI, diffusion-weighted imaging; DCE–MRI, dynamic contrast-enhanced MRI; HNSCC, head and neck squamous cell carcinoma; HPV, human papilloma virus; MRI, magnetic resonance imaging; PKM, pharmacokinetic models; ^1^H-MRS, proton magnetic resonance spectroscopy; RT, radiotherapy; ROI, region of interest; K^trans^, the volume transfer constant between the blood plasma and extracellular extravascular space
